# Plain Radiography May Be Safely Omitted for Selected Major Trauma Patients Undergoing Whole Body CT: Database Study

**DOI:** 10.1155/2012/432537

**Published:** 2012-07-15

**Authors:** Sarah Hudson, Adrian Boyle, Stephanie Wiltshire, Lisa McGerty, Sara Upponi

**Affiliations:** ^1^Clinical School, Cambridge University, Cambridge CB2 2QQ, UK; ^2^Cambridge University and Emergency Department, Cambridge University Foundation Hospitals, Cambridge CB2 2QQ, UK; ^3^Emergency Department, Cambridge University Foundation Hospitals, Cambridge CB2 2QQ, UK; ^4^Department of Academic Radiology, Cambridge University Foundation Hospitals, Cambridge CB2 2QQ, UK

## Abstract

*Introduction*. Whole body CT is being used increasingly in the primary survey of major trauma patients. We evaluated whether omitting plain films of the chest and pelvis in the primary survey was safe. We compared the probability of survival of patients and time to CT who had plain X-rays to those who did not. *Method*. We performed a database study on major trauma patients admitted between 2008 and 2010 using data from Trauma, Audit and Research Network (TARN) and our PACS system. We included adult major trauma patients who has an ISS of greater than 15 and underwent whole body CT. *Results*. 245 patients were included in the study. 44 (17.9%) did not undergo plain films. The median time to whole body CT from the time of admission was longer (47 minutes) in patients having plain films, than those who did not have plain films performed (30 minutes), *P* < 0.005. Mortality was increased in the group who received plain films, 9.5% compared to 4.5%, but this was not statistically significant (*P* = 0.77). *Conclusion*. We conclude that plain films may be safely omitted during the primary survey of selected major trauma patients.

## 1. Introduction

Trauma is the leading cause of death in young people in the UK [1, 2], and 36 life years are lost per trauma death on average [3]. In addition to mortality, trauma is responsible for a hefty morbidity burden; for every death, two survivors suffer disability [4]. Initial management is therefore crucial, particularly as the majority of trauma deaths occur within the first hour of injury [5].

The diagnostic gold standard for major trauma patients on admission to the Emergency Department (ED) is whole-body computerised tomography (CT) [1]. The development of multislice CT has made CT a rapid and accurate diagnostic tool [6]. CT is becoming increasingly accessible in the UK since its introduction in the 1970s [7, 8]. The quality of CT as a diagnostic tool has been proven in several studies and it has revolutionised the management of major trauma patients [5, 6]. The National Confidential Enquiry into Patient Outcome and Deaths (NCEPOD) advises the use of CT for major trauma patients [1], as does the most recent guidance from the Royal College of Radiologists [9], which recommends that multislice CT is adjacent to or in the emergency room. 

The ATLS (Advanced Trauma Life Support) version 8 guidance advocates plain radiography of the chest and pelvis as part of the primary survey of the major trauma patient [5]. This is usually performed before transfer to CT or theatre [10]. Whether plain radiography adds to diagnosis in this situation is uncertain [11]. Anecdotally, we have observed senior emergency physicians omitting plain films if a CT could be acquired promptly. Guidance from Royal College of Radiologists, inseverelyinjured patients, recommends that where definitive imaging such as CT is deemed necessary, less accurate imaging such as plain X-rays should be omitted as they are irrelevant [9]. There is little evidence to support or refute this recommendation. 

A postulated limitation of whole-body CT is increased time taken for transfer to CT compared to time taken for conventional radiography. Conventional radiography can be performed in the ED and requires no transfer [12]. CT may have low specificity for serious injury [13]. Conversely, plain X-rays are less sensitive, may waste time and causes a small amount of unnecessary radiation exposure if performed in addition to whole-body CT [13]. 

We aimed to evaluate whether omitting plain films of the chest and pelvis in the primary survey was safe. We aimed to establish the efficacy of conventional radiography performed in addition to whole-body CT. We aimed to quantify the delay to CT associated with plain films. We also aimed to see whether mortality was different in patients who underwent plain films compared to those who underwent whole-body CT alone.

## 2. Methods

We conducted a single-centre database study. We used our local Trauma Audit and Research Network (TARN) data at Addenbrooke's Hospital. All trauma units in the UK are expected to submit data to this network. The data is entered by trained data entry clerks who review all trauma patient's written and electronic records after death or discharge. The TARN database includes all trauma patients with an Injury Severity Score (ISS) greater than 8. It has had high quality TARN data capture since 2008. We examined data from TARN from January 2008 to December 2010. We included patients if they had an ISS greater than 15, were adult (over 16 years of age), and had undergone a whole-body CT. We excluded secondary transfers from other hospitals. Our outcome was death within thirty days. Our CT scanner is not within our emergency department, but within 50 metres of the resuscitation room. 

Our patients undergo whole-body multislice CT which is defined as imaging of the thorax and upper abdomen in arterial phase and imaging of the abdomen and pelvis at portal phase. Detailed imaging information was retrieved from the hospital's Picture Archiving and Communication System (PACS). We obtained the time that the CT was performed from the CT scan information. We categorised reports as “normal” or “abnormal” for the plain pelvic and chest X-rays and CT scans. Abnormal was defined as any finding which was likely to be due to the trauma for which the patient was admitted. Imaging findings of past injuries or incidental pathology were ignored. 

## 3. Statistical Analysis

A descriptive data analysis and outcome analysis was performed. Stata version 7 was used for statistical analyses. We report performance characteristics of plain films of the chest and pelvis, compared to CT. 

A Pearson's chi-squared median test of equality was carried out on the data to determine the statistical significance of the time delay to whole-body CT if plain films were performed or not. Hypothesis testing with the logrank test for equality of survivor functions was used to compare the time to CT. Kaplan-Meier survival estimates by plain films were calculated for time to CT.

We used Fisher's exact test or the chi-squared test for categorical data. There was no data to guide our study size, so we analysed the largest possible data set that had high quality data capture. 

## 4. Results

During the 3-year period (2008–2010), 493 trauma cases were identified from the TARN database with ISS of 15 or greater. 26 cases were excluded because they were under 16 years of age. 222 cases had not undergone whole-body CT. 245 cases were analysed. One case went to theatre for damage control surgery before CT scan. 

Table 1 shows the demographic characteristics of the patients. The majority of our patients were male and had suffered blunt trauma. There was a low proportion of penetrating trauma. The patient characteristics were broadly similar in the two groups. 

Table 2 shows the time from arrival to CT scan. Performing plain films was associated with a longer time to CT. 

Table 3 shows the performance characteristics of pelvic and chest plain X-ray when compared to whole body CT. 

**Figure 1:**
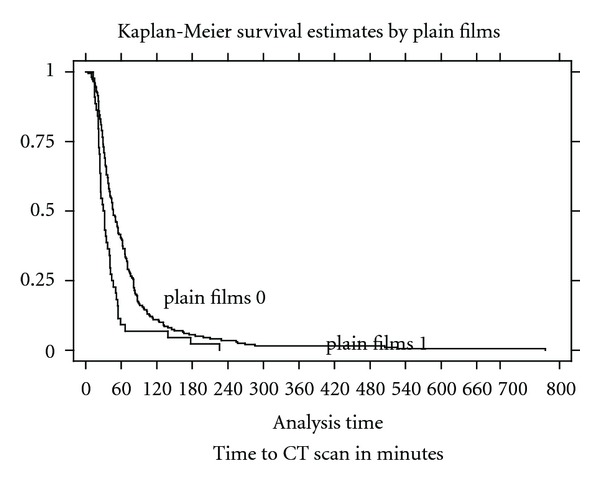
Kaplan-Meier plot showing time to CT if plain films were performed or not.

Patients who underwent plain films in the emergency department had a greater delay to CT scan than those who did not.  Figure 1 shows Kaplan-Meier time to CT by plain films. This data shows that plain films significantly increase the time from arrival in the ED to whole-body CT.

Analysis of mortality data for the plain X-ray and whole-body CT of only groups (Table 2) using Pearson's chi-squared test showed that patients who underwent plain films were more likely to die, 4.5% versus 9.5%, but this was not statistically significant: Fisher's exact test *P* value is 0.77.

## 5. Discussion

We have evaluated the efficacy of plain films in the initial management of major trauma. We found that performing plain films was associated with a longer delay to CT and that this was associated with an increased mortality, but this was not statistically significant. We have found that the chest X-ray has limited sensitivity for detecting abnormalities compared to CT. Pelvic X-rays have better sensitivity and specificity. 

The key findings are that performing plain X-rays significantly delays time from admission to whole-body CT and that plain films add little meaningful information. Mortality was increased in the group who received plain X-ray in addition to whole-body CT, 4.5% versus 9.5%, but this was not statistically significant (*P* = 0.38). 

The evidence base for the superior performance of multislice CT over conventional radiography as a diagnostic tool in the setting of major is well established. We found that pelvic and chest X-ray, are less sensitive, 94.2% and 59.9%, respectively, compared to CT, for injury than imaging of the pelvis and chest by whole-body CT. A question is whether injuries diagnosed on whole-body CT but not on X-ray are clinically significant and require treatment. Smith et al. found that CT was more sensitive but significantly less specific, but that CT did diagnose clinically important injuries which would otherwise have been overlooked in a significant number of patients [7].

More patients who underwent plain films died; the difference in recorded probability of survival between the two groups was not significant (*P* = 0.77). This outcome along with the existing evidence in support of the superior performance characteristics of CT over plain X-ray suggests that use of plain X-ray for pelvic and chest imaging is of little clinical value and can cause an unnecessary delay to diagnosis. The Royal College of Radiologists states that “delay is deterioration, disability and death” [9]. This is corroborated by Huber-Wagner et al. [14] who found that mean time from trauma room admission to whole-body CT was significantly shorter and survival was significantly greater for whole-body CT compared to non-whole-body CT. This contradicts the idea that portable radiography is faster as it can be carried out in the resuscitation room compared to CT which requires transportation to the radiology CT suite [14]. In addition, The National Confidential Enquiry into Patient Outcome and Deaths (NCEPOD) has advised the use of CT for imaging the major trauma patient [1].

Other recent studies have found an association between inclusion of plain films in the primary survey and morbidity and mortality. Smith et al. [15] found that CT was more sensitive for occult injury than plain X-rays in major trauma patients. 18% of these injuries required immediate intervention [15]. Hilty et al found that conventional X-ray gave a false negative in 4% of cases when compared to CT [11]. Huber-Wagner et al. who found that mean time from trauma room admission to whole body CT was significantly shorter and survival was significantly greater for whole-body CT compared to non-whole-body CT [14].

We suggest that the unnecessary excess radiation exposure imposed by pelvic and chest X-ray imaging in addition to the better quality whole-body CT potentially poses a greater risk than benefit. Pelvic and chest X-rays involve exposure of radiosensitive organs such as the gonads and breast [16]. The radiation dose of conventional radiography is considerably less than that of whole-body CT: a chest X-ray is 0.02 mSv compared to 10 mSv for whole-body CT [17]. 

There are some important limitations to this study. The increased mortality that we saw in our patients may not be related to time to CT, but may be because of increased injury severity. It is notoriously difficult to adjust for injury severity and the increased mortality in patients we saw may reflect an unmeasured confounding effect. TARN does not collect data around comorbidity, and this may be another unmeasured confounder. Our sample size is small, with only 44 cases having no plain films. This study was conducted in a single centre with exemplary access to CT scanning and it is not clear whether these findings can be applied to other hospitals. Worldwide, not all hospitals that receive major trauma patients have easy access to CT. Time to CT can questioned as a meaningful outcome measure. Time to theatre is not well recorded by TARN and there is an increasing tendency for many injuries to be managed nonoperatively or by interventional radiology. Many life threatening injuries do not require operative interventions, but can be usually treated outside the operating room, for example haemothorax. However, mortality was lower in patients who underwent no plain films and the probabilities of survival were approximately similar in the two groups. 

Clinical examination, with or without focused ultrasound for trauma (FAST), is an essential part of the primary survey and must not be overlooked [18]. Examination is specific for serious injuries if not sensitive and may rule out the need for an immediate CT in the trauma patient with a lower ISS [15]. There is evidence that as ISS increases, examination is less accurate. 

Future work should consider validating this work across other instuitions. A high-quality equivalence randomised controlled trial is unlikely to be performed, given the problems of consent in unconscious patients, the low cost of plain films, and the large sample size that this study would require. There are a number of unanswered questions arising from this work. It is not clear whether these findings can be applied to haemodynamically unstable patients; most of our sample was haemodynamically stable. Currently, we obtain plain films on these patients. The role of clinical examination and whether this would influence the use of plain films is also undefined. Our results do not provide any information about the usefulness of low-dose full-body X-rays systems, such as the LODOX system. 

## 6. Conclusion

We have demonstrated that plain films of chest and pelvis are being safely omitted as part of the primary survey of adult major trauma cases in our institution. 

## Figures and Tables

**Table 1:** Demographic data of patient groups: whole-body CT with plain radiographs and whole-body CT without plain radiographs.

	No plain films *n* = 44 (%)	Plain films *n* = 201 (%)	Chi-squared test (*P* value)
Sex			
Female	13 (29.5)	53 (26.4)	0.17 (1 df *P* = 0.68)
Male	31 (70.4)	147 (73.4)	
Age			
16–24	5 (11.4)	49 (24.4)	8.16 (5 df *P* = 0.15)
25–34	4 (9.1)	37 (18.40	
35–44	10 (22.7)	34 (16.9)	
45–54	9 (20.5)	24 (11.9)	
55–64	9 (20.5)	28 (13.9)	
65 + plus	7 (15.9)	29 (14.4)	
Mechanism of injury			
Blow(s)	0 (0)	4 (1.9)	*P* = 0.10*
Crush	1 (2)	2 (1.0)	
Fall less than 2 m	0 (0)	13 (6.5)	
Fall more than 2 m	13 (29.5)	29 (14.4)	
Other	0 (0)	1 (0.5)	
Stabbing	0 (0)	1 (0.5)	
Road traffic collision	30 (68.2)	151 (75.1)	
Trapped at scene			
No	27 (61.3)	137 (68.1)	0.84 (1 df *P* = 0.36)
Yes	17 (38.6)	63 (31.3)	
Initial GCS			
3–8	11 (25)	62 (30.8)	0.63 (2 df 0.63 *P* = 0.73)
9–12	5 (11.4)	22 (10.9)	
13–15	38 (86.3)	117 (58.2)	
Outcome			
Alive	42 (95.5)	182 (90.5)	*P* = 0.38*
Dead	2 (4.5)	19 (9.5)	
Probability of survival (%)			
0–20	0.0 (0.0)	6 (3.0)	*P* = 0.77*
20–40	4 (9.1)	23 (11.4)	
40–60	4 (9.1)	22 (10.9)	
60–80	8 (18.2)	25 (12.4)	
80–100	28 (63.6)	125 (62.2)	

^∗^Fischer's exact test was used here as there were cells with low counts.

**Table 2:** Time from arrival to whole-body CT (minutes).

	No plain films	Plain films	Median test of equality
Median	30	47	Chi^2^ (df = 1) = 12.5 *P* < 0.001
Mean (standard deviation)	42.1 (41.7)	69.7 (83.7)	

**Table 3:** Performance characteristics of plain films compared to CT.

Performance characteristic	Pelvis (*n* = 44)		Chest (*n* = 201)	
		95% CI		95% CI
Sensitivity	94.9	85.8–98.9	59.9	52.0–67.4
Specificity	74.4	63.6–83.4	95.7	78.1–99.9
Negative predictive values	95.3	86.9–99.0	24.7	16.2–35.0
Positive predictive value	72.7	61.4–82.3	99.0	94.6–100
Likelihood ratio of a positive test	3.7	2.4–5.9	13.8	2.4–674.0
Likelihood ratio of a negative test	0.07	0.01–0.2	0.4	0.4–0.7
